# The Cardiomyopathy Lamin A/C D192G Mutation Disrupts Whole-Cell Biomechanics in Cardiomyocytes as Measured by Atomic Force Microscopy Loading-Unloading Curve Analysis

**DOI:** 10.1038/srep13388

**Published:** 2015-09-01

**Authors:** Thomas Lanzicher, Valentina Martinelli, Luca Puzzi, Giorgia Del Favero, Barbara Codan, Carlin S. Long, Luisa Mestroni, Matthew R. G. Taylor, Orfeo Sbaizero

**Affiliations:** 1 Department of Engineering and Architecture, University of Trieste, Via Valerio 2, 34127, Trieste Italy; 2 International Center for Genetic Engineering and Biotechnology, Area di Ricerca, Padriciano 99, 34149 Trieste Italy; 3 Department of Food Chemistry and Toxicology, University of Vienna, Waehringer Str. 38 A, 1090 Vienna; 4 University of Colorado Cardiovascular Institute, University of Colorado Denver AMC, 12700 East 19th Ave. Aurora 80045, Colorado, USA

## Abstract

Atomic force microscopy (AFM) cell loading/unloading curves were used to provide comprehensive insights into biomechanical behavior of cardiomyocytes carrying the lamin A/C (LMNA) D192G mutation known to cause defective nuclear wall, myopathy and severe cardiomyopathy. Our results suggested that the LMNA D192G mutation increased maximum nuclear deformation load, nuclear stiffness and fragility as compared to controls. Furthermore, there seems to be a connection between this lamin nuclear mutation and cell adhesion behavior since LMNA D192G cardiomyocytes displayed loss of AFM probe-to-cell membrane adhesion. We believe that this loss of adhesion involves the cytoskeletal architecture since our microscopic analyses highlighted that mutant LMNA may also lead to a morphological alteration in the cytoskeleton. Furthermore, chemical disruption of the actin cytoskeleton by cytochalasin D in control cardiomyocytes mirrored the alterations in the mechanical properties seen in mutant cells, suggesting a defect in the connection between the nucleoskeleton, cytoskeleton and cell adhesion molecules in cells expressing the mutant protein. These data add to our understanding of potential mechanisms responsible for this fatal cardiomyopathy, and show that the biomechanical effects of mutant lamin extend beyond nuclear mechanics to include interference of whole-cell biomechanical properties.

Assessing the effects of specific biomechanical forces on cells expands our understanding of disease pathology and can accelerate the development of biomedical applications such as tissue engineering. For cells to be effective in their tissue-specific roles, they need to have distinct mechanical properties such as elasticity in order to survive mechanical stress and to convert forces into biochemical signals, a phenomenon called mechano-transduction. In addition, nuclear elasticity has been proposed to be a regulator of force transduction on chromatin and genetic expression^1^,^2^. Therefore, a change in either cell or nuclear elasticity to non-physiological values disrupts cellular homeostatic mechanisms and may result in a pathological state leading to a disease: examples include increased stiffness in breast cancer^3^ and bladder cancer cells^4^. Among the tools available in mechano-biology to understand how cells respond to applied forces, Atomic Force Microscopy (AFM) provides the unique opportunity to directly examine the nanoscale structure of cell membrane surfaces, as well measure the mechanical properties of living cells and assess their real-time changes^5^.

In this study we used AFM to measure the nuclear elasticity (Young modulus) and the cell biomechanical behavior during loading and unloading cycles in a single neonatal rat ventricular myocyte (NRVM) model carrying the lamin A/C gene (*LMNA*) D192G mutation^6^. Lamins are type V intermediate filaments found in the nucleus of virtually all cell types and are a key component for providing the integrity of the cell nuclear envelope^7^,^8^ and *LMNA* mutations cause at least 12 distinct diseases (*laminopathies)*, including severe muscle dystrophies and dilated cardiomyopathies^8^. The D192G LMNA mutation, which affects a highly conserved residue of the α-helical coil 1B domain predicted to alter lamin assembly, was found to be associated with a severe dilated cardiomyopathy phenotype^6^. In patients’ cardiomyocytes, the mutation caused dramatic morphological alterations, including a complete loss of the nuclear envelope and chromatin disorganization which may cause altered transcriptional regulation and nucleocytoplasmic transport.

Understanding how *LMNA* mutations impact the mechanical properties of the nucleus and cell as a whole in a cardiomyocyte model might provide novel insights into the underlying mechanisms of these diseases. Past efforts to study the mechanical properties of the nucleus in laminopathies have used indirect measurements such as micropipette aspiration and imaging^1^ or computational modeling^9^. However, AFM provides a more advanced and direct approach to the study of nuclear biomechanics by allowing the observation and manipulation of biological surfaces in their native environment at a very high spatial resolution, and relying on a signal-to-noise ratio superior to that of optical microscopic techniques^10^.

## Results

### Analysis of LMNA expression in cardiomyocytes

**Figure 1:**
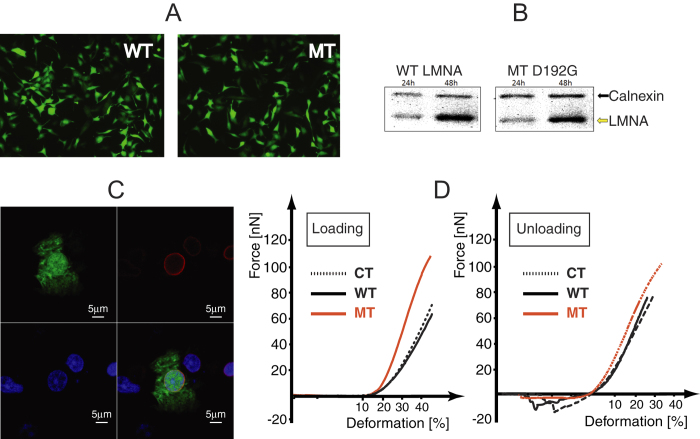
(**A**) EGFP and human LMNA expression detected by fluorescence light microscopy 24 hours post-infection with adenoviral WT and MT NRVM’s constructs. (**B**) Expression of the transduced human LMNA protein detected by human specific anti-LMNA antibody by western blot: human LMNA and rat Calnexin at 24 and 48 hours post-infection. (**C**) Indirect immunofluorescence showing co-localization between GFP (green, upper-left panel) and human LMNA (red, right-upper panel) signals in D192G LMNA NRVMs, transduced by adenoviral bicistronic GFP-LMNA construct. The nuclei are stained in blue (TOTO3, lower-left column). The fluorescence is represented in three channels. Bars: 5 μ. As seen in the merged panel (lower-right), human LMNA is perfectly co-localized in the area of the nuclear wall. (**D**) AFM loading-unloading curves for CT, WT *LMNA* and MT *LMNA* D192G.

Neonatal rat ventricular myocytes (NRVMs) were isolated and enriched (> 90% purity) over non-myocytes as previously reported^11^,^12^,^13^,^14^ and subjected to infection with an adenoviral construct carrying either the wild type or the mutant D192G *LMNA A* cDNA as well as the Enhanced Green Fluorescent Protein (EGFP) which can be used to identify LMNA expressing cells (described in detail in the Methods section)^15^,^16^,^17^,^18^,^19^. NRVMs were infected on culture day 1 and the expression of both EGFP and human LMNA (wild-type and mutant) examined after 24 and 48 hours. As indicated in Fig. 1A,B, although EGFP expression was always detected visually by 24 hour of infection, the expression of the LMNA proteins, as determined by Western blotting with a human-specific anti-lamin A antibody, appeared at 24 hours and was clearly expressed at 48 hours. Furthermore, as shown in Fig. 1C, immunofluorescence confirmed the localization of the exogenous human LMNA (in red, right panels) in the nuclear wall of D192G LMNA NRVMs, when transduced by adenoviral bicistronic GFP-LMNA constructs. Increases in cell-turnover or senescence during the time in culture or in response to adenoviral infection were not observed (data not shown).

### AFM force-deformation curves

Two different AFM cantilever tips were used to precisely apply a compression force normal to the nucleus: (i) a sharp silicon nitride tip or (ii) a polystyrene microsphere with a diameter of about 10 μm coated with a gold layer. We used both since they provide different information: the sphere evaluates virtually the whole nucleus elasticity and can be used to assess potential tip-membrane adhesion forces, while the sharp tip provides information about the local elasticity it also provides qualitative information on nuclear fragility (slope change or drops in the loading curve). Figure 1D shows AFM force-deformation curves collected with the spherical tip during loading and unloading, for control (CT), wild-type (WT) and mutant (MT) cells, respectively. All curves displayed a smooth and nonlinear deformation profile without irregularities or stress peaks, indicating their elastic nature and suggesting no structural failure of the nuclear envelop. These curves provide some significant information: (*i*) the first part of the loading curve provides nuclear elasticity (Young Modulus), (*ii*) the loading curve also provides the maximum load needed to deform the cell, and (*iii*) the area under the deformation curves, during the unloading cycle, reflects cell adhesion behavior.

#### (i) The nuclear Young Modulus is increased in LMNA D192G NRVM

**Figure 2:**
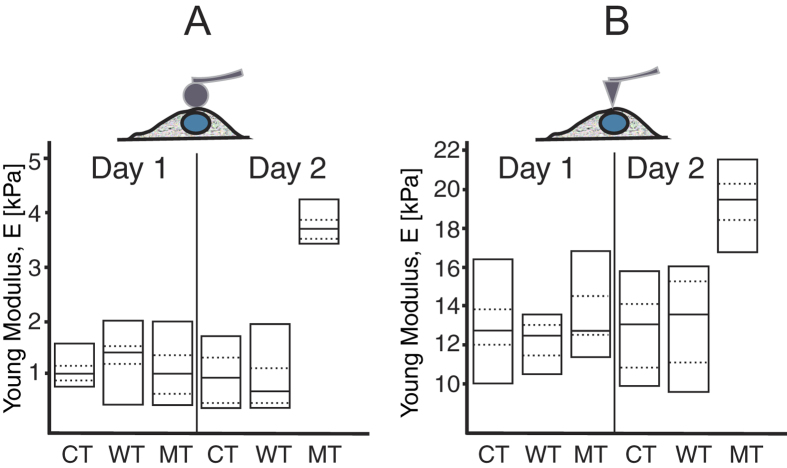
(**A**) Young modulus of the nucleus obtained with a sphere tip, after 24 and 48 hours from adenoviral infection. The plots show the difference in elasticity for the uninfected control cell (CT), WT *LMNA* and MT *LMNA*-D192G, respectively. Mean (black line) values for each condition are shown within the boxes. (**B**) Young Modulus of the nucleus obtained using a sharp tip.

Figure 2A shows the elasticity results (assessed with a sphere tip) for uninfected control, wild type and mutant D192G-expressing cells. Data shown in Fig. 2A are those obtained after 1 and 2 days from the adenoviral infection and indicate that CT and WT elasticity remained substantially constant over the 2 days of observation. In contrast, the Young’s modulus in cells expressing the *LMNA* D192G mutation began to diverge from both CT and WT after 24 and was significantly higher than that of both CT and WT at 48 hours.

The elasticity data acquired with the sharp tip cantilever (Fig. 2B) showed a similar trend, although nuclear elasticity values were about five to six times higher than those obtained with spherical probes.

#### (ii) Mutant D192G lamina becomes brittle

The sharp tip has allowed us to highlight a clear difference between the nuclear fragility of the different LMNA expressing NRVM’s. For MT NRVM’s, 65% of the cells showed either an abrupt change in the slope or a sudden drop in the loading curves: this behavior is typical of nuclear membrane yielding and therefore a brittleness (“fragility”) of the nucleus that is not seen in either CT or WT cells where less than 5% of cells examined showed this behavior during the loading cycle. Although we did not observe signs of permanent cellular injury such as the development of an abnormal morphology, detachment from the substrate, or cytoplasmic vacuolization, these results are in line with previous reports suggesting increased nuclear fragility^19^.

#### (iii) LMNA D192G causes a decrease in cell membrane adhesion

Adhesion areas recorded during the end of the unloading cycle show that CT and WT cells exhibit similar adhesion areas while MT NRVM’s show virtually no adhesion area (Fig. 1D).

### Influence of the Lamin mutation in the cytoskeletal organization of NRVM

**Figure 3:**
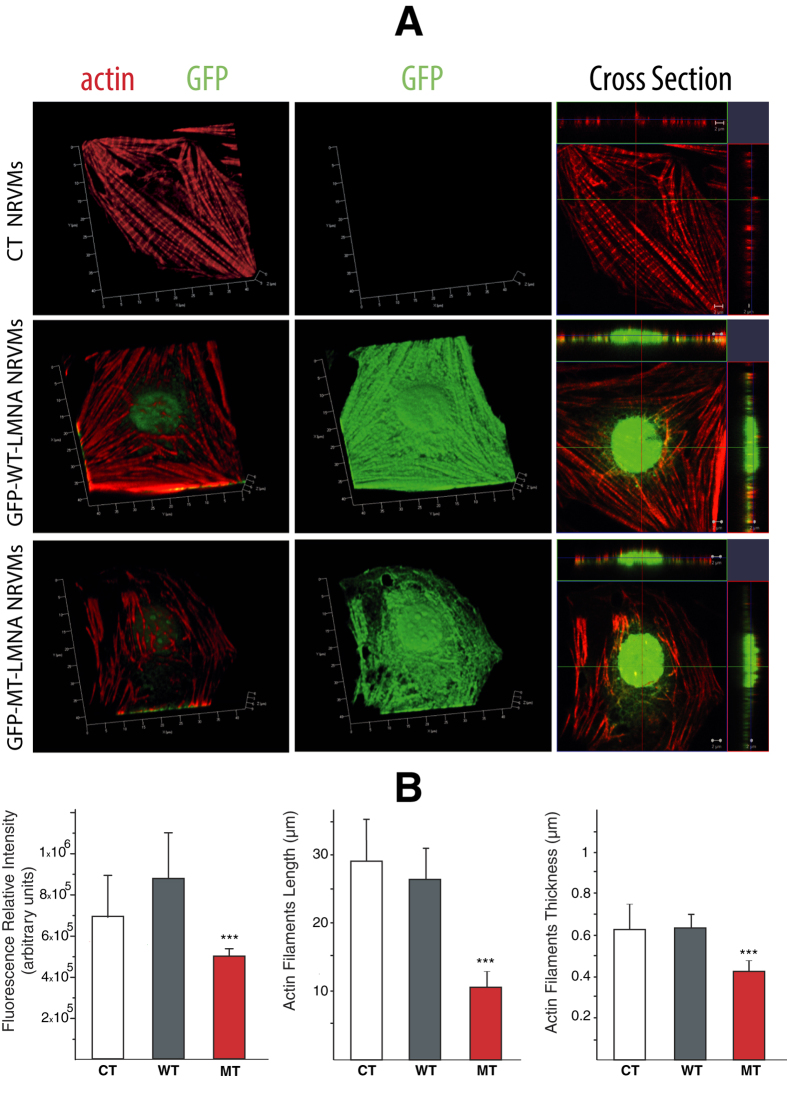
D192G LMNA seems to induce actin network alteration in NRVMs: (**A**) 3D reconstruction of the appearance of actin cytoskeleton (red) in NRVMs in control conditions and after infection with WT and MT-GFP LMNA. NRVMs infected with MT-GFP LMNA displayed a lower actin fibers density into the cytoskeleton and the presence of blebs on nuclear membrane clearly visible in a 3D reconstruction of a Z-stack acquired in a range of 9.81 μm. (**B**) Quantification of red fluorescence relative intensity (n = 3 optical fields for each experimental condition), and appearance of labeled actin filaments length (CT n = 38 fibers; WT n = 40 fibers; MT n = 67 fibers) and thickness (CT n = 67 fibers; WT n=86 fibers; MT n = 82 fibers). ***Student t-Test significant difference in comparison to controls (CT) at *p* < 0.001. Data show a significant decrease of fluorescence relative intensity, actin filaments length and actin filaments thickness in MT NRVM compared to CT and WT cells.

In order to investigate if the alteration of the mechanical/adhesion properties of the mutant cardiomyocytes could be due to an alteration of the cytoskeletal structure, laser scanning microscopy confocal images were acquired. In control conditions, the actin cytoskeleton appeared to be highly organized and homogeneously distributed (Fig. 3A, red). Similarly, in cardiomyocytes expressing WT lamin actin cytoskeleton seemed organized and perfectly integrated with both the GFP and the WT lamin proteins. On the contrary, mutant cells, morphologically identified by the presence of the typical bleb in the nuclear lamin, seemed to be characterized by an altered cytoskeletal organization. This alteration in the cytoskeletal organization was particularly evident in the peri-nuclear region, as clearly highlighted by the relevant cross-section of the 3D reconstruction of the z-stack (Fig. 3A). Furthermore, a significant decrease of (i) red fluorescence relative intensity, (ii) actin filaments length and (iii) actin filaments thickness were confirmed by quantitative measurement of the actin amount and morphology in MT NRVM compared to CT and WT cells (Fig. 3B, ***student t-Test significant difference in comparison to controls (CT) at *p* < 0.001).

**Figure 4:**
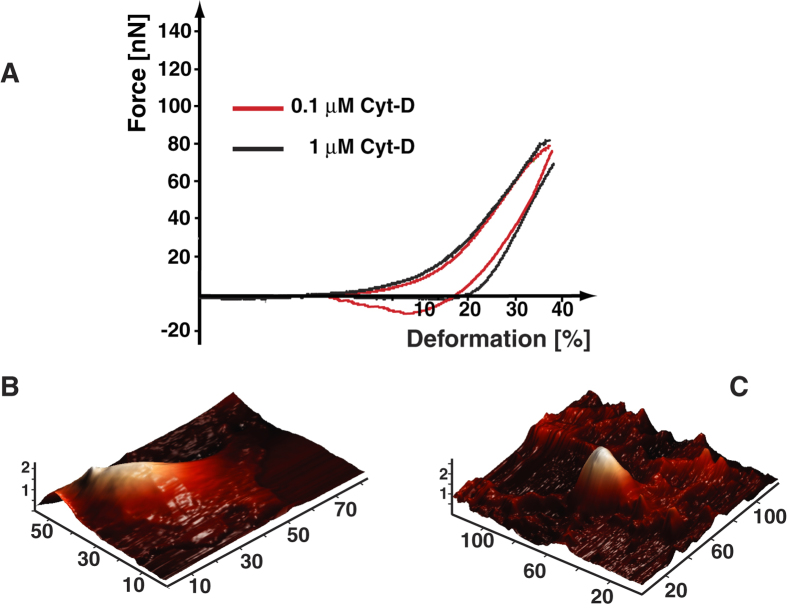
Effect of actin filaments derangements with Cytochalasin D in NRVM: (**A**) Loading/unloading curves for control cells after administration of 0.1 and 1 μM of Cytochalasin D. (**B**) 3D-Cell morphology reconstruction obtained from AFM scan in uninfected non-treated cells and, (**C**) uninfected NRVM’s subjected to Cytochalasin D.

Moreover, in order to further test the hypothesis that the alteration of the mechanical properties of cardiac myocytes carrying Lamin D192G mutation could be due to an alteration of cellular cytoskeleton, additional AFM experiments with the cytoskeleton-disrupting agent Cytochalasin D (Cyt-D) were performed. Cyt-D is known to produce depolymerization of the actin filaments causing a dramatic alteration in cellular adhesion properties^20^,^21^. Indeed, our AFM measurements were able to detect differences in the adhesion areas recorded on NRVM cells treated with Cytochalasin D compared to control: as shown in Fig. 4A, at a concentration of 1 μM Cytochalasin, actin disruption clearly modified the adhesion area, producing a behavior similar to that seen in MT *LMNA* D192G cells. Figure 4B is a 3D reconstruction of an AFM scan of an untreated NRVM, while Fig. 4C a Cytochalasin-treated NRVM: the latter shows cytoskeletal morphological changes such as cytoplasmic condensation with formation of dense aggregates. Despite the clear changes in the cytoskeleton of cells treated with Cyt-D, the nuclear elasticity remained unchanged (1.12 × 10^3^ ± 7.7 × 10^2^ Pa for the CT and 1.09 × 10^3^ ± 5.6 × 10^2^ Pa for the CT + 1 μm Cyt-D, respectively, *P* = 0.64).

Furthermore, to verify the adhesion behavior reproducibility, we performed 10 repetitions on the same cell (CT-NRVM’s, MT-NRVM’s and Cytochalasin-D treated NRVM’s, respectively). These studies showed that adhesion area was always present for CT cells, while both mutant and the Cyt-D treated cells, after the first cycle the adhesion area was already strongly reduced and, within the statistical reproducibility of AFM test, the area remained very small also in the subsequent cycles. Concentrations of 0.25 μM and 0.5 μM of Cyt-D also cause, in control NRVMs, a drastic reduction of adhesion between AFM tip and cell membrane with no rounding of cells at any concentration tested.

## Discussion

### AFM force-deformation curves

AFM cell loading-unloading curves contain several information about both short (within the nucleus) and long (between the nucleus and cell membrane via the cytoskeleton)-range interactions. In fact they represent a basis not only for assessment of elasticity of the nucleus itself, but also for evaluating overall cell deformation and nuclear-cytoplasmic interactions. As the results have shown, three distinctive features can be derived from these curves (Fig. 1D): (*i*) the nuclear elasticity, (*ii*) the maximum load, and (*iii*) the area under the deformation curves during the unloading cycle reflecting de-adhesion of the AFM sphere from the cell membrane. Analysis of this latter phase may provide insight into several intrinsic properties in the process of detachment which include: the maximum separation force (in absolute values), the separation energy i.e. the area enclosed by the curve and the zero force axis, and a number of small detachment “steps” which could be related to the number of bonds broken between the AFM sphere and membrane adhesion proteins^22^.

Our data indicate that cardiomyocytes carrying the *LMNA* D192G mutation have an increased nuclear Young modulus compared to control NRVMs as well as NRVMs expressing wild-type *LMNA*. Since NRVM’s expressing wild-type *LMNA* displayed similar stiffness to controls, the higher stiffness in *LMNA* D192G NRVM’s is likely to be due to the mutant LMNA protein. Furthermore, we found that the elasticity of MT nuclei varied over time (from day 1 to day 2), due to the level of expression of the transduced gene whereas stiffness of both CT and WT remained stable.

For small deformations, a cell nucleus can be modeled either as^23^:

**(1) A sphere filled with an incompressible fluid**, which during compression retains a constant volume due to the impermeability of the boundary. The balloon shell (nucleus) balances the compressive pressure by stretching therefore increasing its surface area. In this case the force (F) and nucleus deformation (ε) may be correlated:


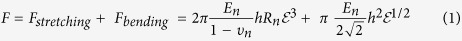


where *R*_n_ and *h* are the radius of the uncompressed nucleus and its membrane thickness, respectively, and *E*_n_ and *ν*_n_ represent the Young’s modulus and Poisson ratio, respectively. The ratio between the bending and stretching terms can be calculated using


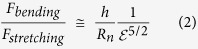


Given that the nuclear membrane thickness (*h*) is typically 20–30 nm, while the cell radius is around 3 μm, this ratio is quite small. Therefore, one can neglect the small bending deformation term, and Equation 1 becomes:


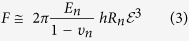


(2) **A sphere filled with fluid but with a permeable skin**. During AFM compression, fluid may be squeezed out, at which point the membrane stretching term becomes insignificant. The small bending of the spherical membrane can be estimated as:


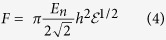


In the paper by Lulevich *et al.*^23^ this behavior was related to that of dead/dying cells.

(3) **A hard rubber ball.** In this case, the force varies with the deformation as^24^:


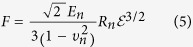


Lulevich *et al.*^23^ showed that this relation between force and deformation can be used for stiff cells like those fixed.

**Figure 5:**
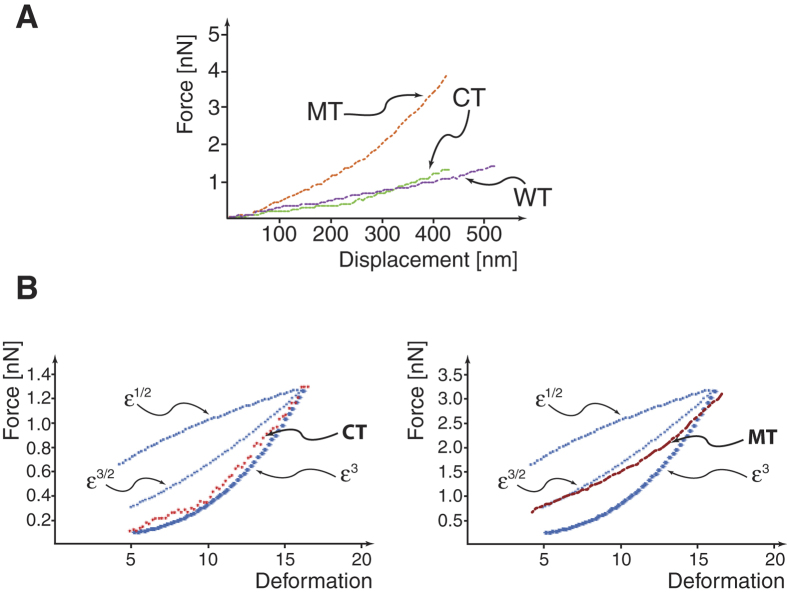
(**A**) AFM force-deformation curves for CT, WT and MT cells respectively. (**B**) CT and MT deformation behavior. CT follows quite well a cubic relationship with respect to deformation (ε^3^) while MT cells show a relationship close to ε^3/2^.

In our case, if the AFM vertical displacement up to 500 nm is analyzed, MT cells display a behavior different from that of both MT and WT cells (Fig. 5A). None of the cells considered follow a relation with (ε^1/2^), CT and WT cells follow quite well a cubic relationship with respect to deformation (ε^3^) while MT cells show a relationship close to ε^3/2^ (see Fig. 5B) confirming also in this way that the MT nucleus is stiffer.

**Figure 6:**
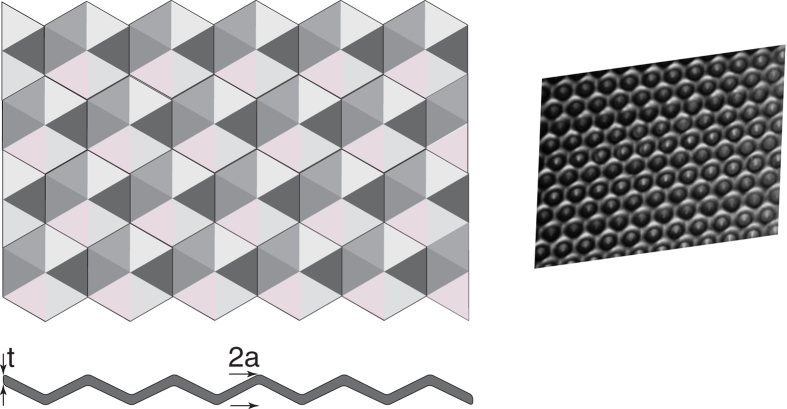
This cartoon shows a structure with dimples and the geometrical parameters used to calculate its bending stiffness. Our biomechanical data suggests that the *LMNA* D192G mutation may change the meshwork shape and therefore its mechanical properties.

In the literature, data regarding elasticity of cellular nuclei are quite divergent, with values ranging from 18 Pa to more than 45 kPa^25^,^26^,^27^,^28^, disparities which are likely due to a variety of factors such as cell type, measurement techniques and conditions, length scale of interest, and also interpretation methods. Moreover, changes in nucleus size, morphology, uniformity, and cytoskeleton complexity could alter the distribution of the stress applied to the nucleus, thereby changing its elasticity. For instance, greater contact between the nucleus and cytoskeleton (such as higher density of SUN1 proteins) could alter the stress in the nucleus, resulting in a different nuclear elasticity^29^. Moreover, we believe that the difference between data assessed with either sphere or sharp tips are due to the fact that the latter describe elasticity at a very focused point on the nuclear envelope while the spherical probe assesses the mechanical properties of the whole nucleus. The dependence of elasticity on probe shape has been previously reported for alveolar epithelial cells where elasticity was two times higher when examined with sharp tips^30^, and macrophages showed five times higher elasticity compared to data obtained with a spherical probe^31^. The structure of the nuclear lamina, a meshwork (a two-dimensional network of orthogonal lamin filaments)^7^ plays a critical role in maintaining the structural integrity of the nucleus. Dahl *et al.*^1^ suggested a natively compressed lamina network, which acts as a “shock-absorber” with elastic extensibility but a nearly incompressible envelope, forming an extremely robust, flaw-tolerant, material. Qin and Buehler^32^ showed that the lamin mesh is a flaw-tolerant material since, its failure strain is largely insensitive to the presence and size of internal defects, and that the failure strain approaches a constant value even as the defect size grows. Therefore, the “healthy” nuclear lamina is a reliable structure that efficiently protects genetic material from external forces and deformation applied on the cell, unless there are genetic modifications that lead to structural changes in lamin organization and affect its mechanical property causing increased susceptibility to injury. In our case, the increased nuclear stiffness of the mutant cells, and the contemporaneous evidence of nuclear membrane yielding and therefore higher brittleness was not exhibited in either CT or WT cells. These observations lead us to hypothesize that the *LMNA* D192G mutation might have changed the meshwork shape and therefore its mechanical properties. It is noteworthy that AFM probe contact conditions have a minimal effect on the effective stretching stiffness of the nuclear envelope, while they alter its bending stiffness significantly^33^. From a structural point of view, bending stiffness is proportional to (E × I_0_) where E is the Young modulus and I_0_ is the second moment of area of a flat sheet, equal to:


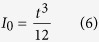


where t = sheet thickness. In the case of a corrugated (dimpled) sheet (Fig. 6), the second moment of area is equal to:


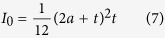


Therefore, the shape factor (Φ_b_) for failure in bending is:


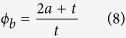


Equation 8 predicts that a grid of dimples would provide a large gain in stiffness but would change its failure mechanism: whereas a flat structure can yield when bent, dimpled sheets can fail by a local buckling mode and therefore be more brittle. The hypothesis that a change in lamin shape explains both its change in elasticity and its brittleness needs future experimental validation by complementary imaging techniques. Unfortunately, only a few parts of lamin (intermediate filaments) domains of its dimer structure have been crystallized and their atomic structure identified based on x-ray diffraction experiments^34^,^35^,^36^,^37^, and there are persistent challenges in identifying the remaining parts of its structure.

### LMNA D192G decreases cell membrane adhesion

Measuring the force curves on cardiomyocytes, we observed a peculiar behavior, possibly related to the ability of cellular structural elements to rearrange in response to the stimulation of the indenter. Adhesion areas recorded during the end of the unloading cycle show that CT and WT cells exhibit similar adhesion areas while MT NRVM showed virtually no adhesion area (Fig. 1D). In this respect, the characteristic of this behavior made the CT and WT cells look very similar, since in both cases, the cells seemed to adhere to the sphere giving a resistance to indenter detachment.

**Figure 7:**
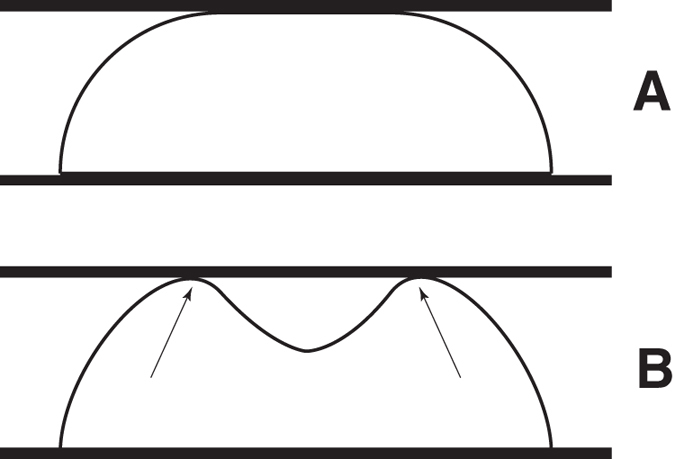
Two possible configurations for the nucleus pressed between two plates. (**A**) the nucleus flattens against the loading surface, or (**B**) as the load increases, a small region buckles (arrows indicate buckled regions).

For both CT and WT cells, the curves that represent the de-adhesion event did not show a smooth shape: therefore, it is likely that they do not originate predominantly from hydrodynamic interactions^38^. Similarly, the detachment-force curves recorded showed an initial, large de-adhesion peak followed by smaller peaks (Fig. 1D). We postulated that both features might be explained taking into consideration bridging of multiple adhesion proteins tails at the cell surface with the nuclear lamina through actin filaments. With this in mind, the different behavior for the mutant cells could be explained by two possible changes in the interaction between the AFM sphere and the cell membrane: (i) the mutant cell could have less surface available for adhesion at the same interface or (ii) less adhesion proteins are available (or less efficient) for adhesion at the sphere/cell interface. The latter hypothesis seems to be the most probable if we analyze the nuclear deformation during the loading cycle. Briefly, as shown in Fig. 7, two situations are conceivable: (i) the nucleus flattens against the loading surface with the profile schematized in Fig. 7A, or (ii) as the load increases, a small region buckles as shown in Fig. 7B (arrows indicate buckled regions). However, the change in elastic energy due to the inversion of a spherical section to create a buckle requires a jump in the value of the force during the loading cycle. As seen in Fig. 1D, the AFM loading curve for the mutant cells do not show any abrupt change in slope, therefore we can rule out the possibility of having less surface available for adhesion.

### Disruption of cytoskeletal proteins replicates defective adhesion behavior in LMNA mutant cells

The transient and local contact between an AFM sphere and a cell surface is a reasonable model for examining the different adhesive property of CT, WT and MT cells. MT cells retraction curves which show little, if any, adhesion suggest that a defective connection exists between the lamin protein within the nucleoskeleton and cell adhesion molecules present on the cell surface. From recent literature^39^,^40^ it is known that there is a continuum from the inner part of the nuclear membrane and the outer part of the cellular membrane, made by several proteins, the Linkers of the Nucleoskeleton to the Cytoskeleton (LINC) complex acting as outside to inside mechano-transducers. Moreover, it has been shown that several proteins, including the LINC complex SUN1/2 and Nesprins, nuclear and cytoskeletal actin, and titin form connections between the lamina nucleoskeleton, and the cytoskeleton^1^,^41^. Ho *et al.*^42^ have recently shown that actin polymerization is altered in *LMNA* mutant cells models (*LMNA* −/− and *LMNA* N195K cells) and that the altered actin may be responsible for the impaired nuclear translocation of MKL1, a cytoskeletal proteins regulator. Thus mechanical properties of both the nucleus and the cell may change in *LMNA* D192G cells when these connections are disrupted and the nucleus undergoes structural reorganization. In particular, actin filaments are connected to the cell membrane through the cell adhesion molecules, and to the nucleus through the nesprin- and SUN-proteins. When cells experience external stress, actin can polymerized to handle these changes. Actin filaments are therefore able to effectively transmit forces to the nucleus and vice versa from the nucleus to the cell membrane, via integrins and the dystrophin complex^41^. The integrity of such a complex network is of vital importance. All the individual elements form one interacting mechanical entity that cannot function properly if any of the composite elements is disrupted. Therefore, in contrast to the classic continuum models of cell mechanics, in which the applied stress will always quickly dissipate and therefore will not be able to transmit any signals to its surroundings, a number of studies suggest that force is directly transmitted from the cytoskeleton to the nucleus, and mutations in the nuclear lamina structure give rise to alterations in the cytoskeleton suggesting that a link must exist between the deregulation of the nuclear lamina, and the effects on the whole cell and affected tissue^41^,^42^,^43^,^44^,^45^.

In our case, confocal microscopy was indeed able to highlight some cytoskeleton morphological modifications: they are presented in Fig. 3. Panel 3A shows the 3D reconstructions of a non-infected, WT and MT cardiomyocyte. It is possible a clear identification of cell phenotype with expression of GFP in both WT and MT and blebs at the nuclear level in the mutant. Most importantly, a different organization of the actin cytoskeleton (red in Fig. 3A) was also visible. At the same time, as shown in panel 3B, we demonstrated that mutant cells displayed an abnormal actin amount and morphology compared to controls. Our confocal microscopy images are consistent with already published data, such as those obtained in mouse models, that highlighted a defective LINC complex as an exclusive condition of laminopathies, leading as a consequence to softer cytoskeleton and reduced adhesion properties^46^. It is also known that a distorted nucleus is a morphological hallmark of cells carrying defective LMNA gene in both human and mouse models. Moreover^47^,^48^ morphometric analysis experiments showed that nuclei of Lmna^L530P/L530P^ cells were particularly prone to mechanical fragility. Our results also showed that MT cells are brittle with a smaller nucleus compared with wild type LMNA cells. Published data^46^ suggested that mechanical alterations found in laminopathic cells could stem from a dysfunctional network between the nucleus and cytoskeleton; our microscopic analyses highlighted that MT LMNA may also lead to a morphological alteration in the cytoskeleton.

Therefore, to test the hypothesis that the mutant nuclear lamina could have altered cellular membrane adhesion properties through alteration of the cytoskeleton, as supporting evidence, we repeated the AFM experiments in presence of Cyt-D. In these conditions, the interaction between AFM tip and cells mirrored that previously shown for the mutant cells.

**Figure 8:**
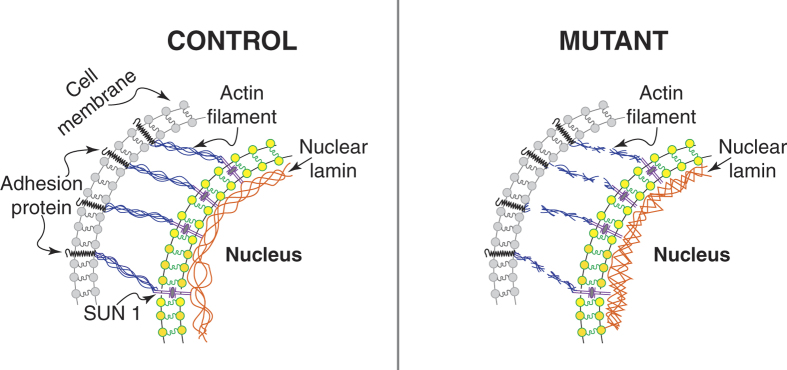
Hypothesis on the role of defective lamin in altering cell adhesion behavior. Speculative cartoon underlying the potential role of mutated LMNA in cell adhesion properties. As recently reported^42^, we hypothesized that lamin mutant cells cause altered actin dynamics and cytoskeletal actin polymerization. The defective nuclear-cytoskeletal connection may lead to adhesion proteins dysfunction, and ultimately to defective adhesion-detachment properties in mutant cells.

In conclusion, to our knowledge, this is the first comprehensive report on abnormalities in AFM-derived mechanical properties of cardiomyocytes expressing a *LMNA* mutation associated with a severe form of familial dilated cardiomyopathy (*LMNA* D192G). These data were derived from analysis of a series of loading and unloading curves of living cardiomyocytes in which expression of both wild-type and mutant proteins were accomplished using an adenoviral expression system that resulted in a time-dependent expression of exogenous protein mirrored by alterations in both stiffness and fragility of the cells. Specifically, *LMNA* D192G transduced cardiomyocytes showed an increase in the nuclear Young modulus and greater nuclear brittleness that were clearly apparent as expression of mutant protein increases. We hypothesize that the concurrent change in both elasticity and brittleness might be explained by a shape change in the nuclear lamin structure going from a simple flat mesh to a more dimpled structure. Moreover, the loading-unloading curves showed: (i) differences in the maximum load required to deform the nucleus where mutant cells required higher loads, (ii) sphere/cell membrane detachment area where mutant cells show almost no detachment area. These results confirm that AFM is a very good tool for cell mechanical perturbation and force measurements. Furthermore, simple models may be utilized to rationalize the observed force-deformation measurements. Similar AFM measurement of the aforementioned parameters in control cells in which the actin cytoskeleton has been chemically disrupted suggest that the alterations in the mechanical properties seen in mutant cells reflects a fundamental defect in the connection between the nucleoskeleton, cytoskeleton and cell adhesion proteins expressed at the cell-surface (Fig. 8). Structural anomalies resulting from mutation of lamin A/C therefore, seems to extend far beyond the nucleus to affect the cytoskeleton as shown in our case for the actin network. Our descriptive experiments clearly demonstrate that there is an adhesion defect in LMNA mutant NRVMs. We speculate that this biomechanical behavior could be driven by defective membrane adhesion proteins, such as integrins, as suggested by previous investigations^19^,^41^,^42^,^43^,^44^,^45^,^46^,^47^,^48^. While a comprehensive functional study of membrane proteins, such as integrins, dystrophin complex proteins, and other specialized complexes such as the desmosome, is beyond the scope of this work, the role of membrane adhesion in laminopathies is intriguing and prompt future investigations.

These insights are a clear addition to our understanding of the mechanism(s) responsible for this fatal cardiomyopathy and suggest that this innovative combination of both cutting-edge engineering and biological techniques may lead to the development of novel approaches to this, and other genetic diseases that affect the mechanical properties of the cell.

## Methods

### Cell cultures

Neonatal rat cardiomyocytes (NRVMs) were isolated and cultured from 1–3 days old Wistar rat pups by enzymatic digestion as previously described with minor modifications^11^,^12^,^13^,^14^. Animal care and treatment were conducted in conformity with institutional guidelines in compliance with national and international laws and policies (European Economic Community Council Directive 86/609, OJL 358, December 12, 1987 and the current Italian law (decree 116/92)). Animals were hosted by the Animal House at ICGEB, Italy, authorized by the Italian Ministry of Health, and breeding conditions and procedures complied with EU guidelines (86/609/CE) and Italian law (decree 116/92). Protocols for tissue explants were performed in accordance with the relevant US and European Union legislation. The entire procedure is in accordance with the regulations of the Italian Animal Welfare Act, with the relevant EU legislation and guidelines on the ethical use of animals and is approved by the local Authority Veterinary Service (Assessorato Sanita’—Servizi Veterinari—Regione FVG). Briefly, ventricles were separated from the atria using scissors and then dissociated in CBFHH (calcium and bicarbonate-free Hanks with Hepes) buffer containing 0.5 mg/ml of Collagenase type 2 (Worthington, Biochemical Corporation), and 1 mg/ml of Pancreatine (SIGMA). Cardiomyocytes were enriched over non-myocytes by a pre-plating step on 100-mm dishes in DMEM containing 10% FBS and 2 mg/ml vitamin B12 (SIGMA). Cardiomyocytes that were either in solution or lightly attached were then separated from the adherent stromal cells by gentle mechanical disaggregation and subsequently were plated at a density of 2 × 10^5^ cells/ml in Dubecco’s modified Eagle medium (DMEM) 4.5 g/L glucose supplemented with 10% horse serum (HS), 5% fetal bovine serum (FBS), 2 mg/ml vitamin B12 and cultured as previously described^11^,^12^. After 12 h, the culture medium was changed and cells were subjected to infection with the relevant adenoviral-*LMNA* constructs infection and subsequently analyzed over the time course described below.

### Adenoviral constructs

Shuttle constructs were generated in human Adenovirus Type5 Dual CCM DNA plasmids containing EGFP gene and human *LMNA* cDNA (Vector Biolabs, Malvern, PA). Constructs were bistronic with the two inserts (*LMNA* and EGFP) driven by two different CMV promoters. The constructs used contained either wild type or mutant *LMNA* D192G cDNA. NRVM’s were infected by adenoviruses at 25 Multiplicity Of Infection (MOI) in serum free medium; 6 h post infection, complete medium was added to cardiomyocytes and the cells were incubated at 37 °C and 5% CO_2_.

### Analysis of LMNA expression

Expression of human and mutant LAMIN-A cDNAs were verified by western blot using anti-laminA antibody, LS-C137845 from Lifespan Biosciences (2401, 4^th^ Ave, Suite 900, Seattle, WA 98121) at dilution 1:1000 for 2 h at room temperature. This monoclonal antibody recognizes human LMNA protein but does not react with rat LMNA. Secondary anti-rabbit-HRP conjugate (Sigma, cat# A0545) at dilution 1:5000 for 1 h at room temperature was used to detect the antigen-antibody complex. Detection was done at days 1 through 5 of infection by ECL Kit (Bioexpress cat# E-1119-20) and visualized in FluorChem system and normalized against levels of calnexin (ab75801 1:5000 dilution).

### Force-deformation curves

An AFM Solver Pro-M (NT-MDT, Moscow, Russia) was used to acquire morphology as well as force-deformation curves. Resonance frequency was verified with the thermal method and was considered acceptable with a variability lower than ± 5% in comparison to the value specified by the supplier (17 kHz). Similarly, also spring constant of the cantilever was verified and considered acceptable between a 25% variability of the value indicated by the supplier (0.08 N/m). Two different AFM cantilever tips were used to precisely apply a compression force normal to the nucleus: (i) a sharp PNP-DB silicon nitride tip (Nanoworld, Neuchâtel, Switzerland) or (ii) a polystyrene microsphere with a diameter of about 10 μm coated with a gold layer. The possible deformation of the polystyrene bead might raise concerns, however the polystyrene Young modulus is about 3 × 10^9^ Pa, much higher than that we measured for our nuclei (around 1 × 10^3^ Pa) and since our goal was to assess the difference in elasticity between a CT, WT and a mutant cell we feel that using strictly the same test protocol will always introduce the same possible, if any, test error. Neither AFM probe was coated with any biological ligand. The AFM was equipped with a “liquid cell” setup for operating on living, intact cells in cell culture medium. Only well-spread and isolated cells were investigated. The AFM indentations were performed on the center region of nucleus of cells plated on the prepared Petri dish to minimize possible sliding during indentation. Cells can be in different states and thus show distinct properties that can be difficult to compare, therefore (i) cells have been prepared following a strict protocol for each cell type. (ii) multiple measurements from a number of different cells were collected to obtain ‘average’ data for each condition and time-point of interest.

Force-deformation curves were plotted as loading force versus relative cell deformation. To quantify the cell compression, relative deformation, ε (cell height change/initial cell height) was used since the cell height varies. The average height of a NRVM was about 2-3 μm. Nuclei were deformed up to 35–40% of their original dimensions. Since cells behave in a viscoelastic manner and appear stiffer at higher AFM approach velocities^49^,^50^,^51^, all cells were deformed at slow speed, 0.5 μm/s. For each experimental condition, data from at least 20 cells were collected for each cell type. The entire test duration was never longer than 45–50 min. to ensure cell health and consistency of data.

### Modeling cells elasticity

Elasticity values can be calculated using various models^52^,^53^,^54^,^55^,^56^,^57^. We used the Hertz-Sneddon model^58^:

for sphere tip:


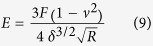


and for the sharp tip


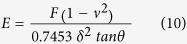


Where E is the Young modulus, F is the load force, ν the Poisson ratio, θ is the tip-cantilever opening-angle, R the sphere radius and δ is the probe penetration into the cell. Since the nuclear elasticity depends on the stage of cell division, we excluded cells with nuclei showing optical mitosis evidence. For the purpose of assessing the Young Modulus we used only the first indentation depth around 200 nm (around 10% deformation) were the behavior is quite linear.

### Immunocytochemistry

Immunocytochemistry of specific CM marker was performed after 2 days of Adenoviral infection on Ad-EGFP-LAMINA-WT, Ad-EGFP-LAMINA-D192G and Non Infected samples. To perform immunostaining, CMs seeded on 0.2% gelatin-fibronectin (SIGMA) coated multichamber slides NUNC, US) were fixed in 4% PFA in PBS for 20 min at room temperature; aldehydes were quenched with 0.1 M glycine in PBS for 10 min at room temperature. Cells were permeabilized with 0.5% TritonX-100 for 1 hour at room temperature. Then, the samples blocked in 2%bovine serum albumine (BSA) were soaked on the followed antibodies: Lamin A/C Rabbit anti-Human Monoclonal (EPR4100) antibody, (LS-C137845 Lifespan Biosciences) at dilution 1:50, and Alpha-Actinin anti-mouse monoclonal (sarcomeric) (EA-53) at dilution 1:100, both O/N at 4 °C in 2% BSA. Anti mouse Alexa Fluor-488 and anti Rabbit Alexa Fluor 594-conjugated secondary antibodies (Invitrogen), 1:500 dilution, were incubated in 2% BSA for 45–60 min at room temperature. All washes were performed in PBS and 0,05% Tween 20. The basal actin cytoskeleton architecture (F-actin) was counterstained with Alexa Fluor® 594 Phalloidin conjugated probe (1:500, 30 min RT; Molecular Probe, Invitrogen). Nuclei were counterstained with 1:20000 dilution of TOTO®-3 ioide for 1 min RT in PBS (Life Technology) and then washed once in water. Finally, the samples were mounted in Vectashield (Vector Laboratories). A first set of pictures were acquired with a Zeiss LSM 510 confocal microscope, representative images were acquired from at least three independent cell preparation. For the purpose of the study a Plan-Apochromat 100X/1.46 objective was used. 3D images were acquired with a Zeiss LSM 710 confocal microscope, equipped with ELYRA system. Representative images were acquired from two different cell preparations that included all the tested conditions: Controls (CT); wild type (WT) and D192G mutant (MT). For the purpose of the study a Plan-Apochromat 100X/1.46 objective was used. In order to obtain complete 3D reconstructions of NRVM, 26 images were acquired for a total extension of 9.81 μm on the z-axes. Quantification of actin filaments relative intensity was performed using imagej software. The appearance of actin filaments after labeling was quantified offline for length and thickness using the Zeiss Zen 2012 SP1 software in three randomly chosen optical fields for each experimental condition.

### Force transmission through the cytoskeleton

For Cytochalasin D (Cyt-D; Sigma Aldrich) treatment, a stock of 5 mg/ml was prepared by reconstituting the drug in sterile DMSO and a working dilution in complete medium was freshly prepared the day of the experiment at the following concentrations: 0.1–0.25–0.5 and 1 μM. A series of experiments showing the lack of biomechanical effects and toxicity of DMSO 1 μl/ml pre- and post-treatment were performed to exclude any effect of the vehicle.

### Data analysis

Elasticity data were analyzed using R, a software for statistical computing and graphics^59^. Data are represented as box plots whose endpoints are the first quartile (Q_1_) and third quartile (Q_3_), with a horizontal line corresponding to the second quartile (Q_2_, median)^59^. Analysis after image quantification was performed with the student t-Test. The differences between groups were evaluated for statistical significance by calculating the *p*-values.

## Additional Information

**How to cite this article**: Lanzicher, T. *et al.* The Cardiomyopathy Lamin A/C D192G Mutation Disrupts Whole-Cell Biomechanics in Cardiomyocytes as Measured by Atomic Force Microscopy Loading-Unloading Curve Analysis. *Sci. Rep.* **5**, 13388; doi: 10.1038/srep13388 (2015).

## References

[b1] Dahl K. N., Kahn S. M., Wilson K. L. & Discher D. E. The nuclear envelope lamina network has elasticity and a compressibility limit suggestive of a molecular shock absorber. J. Cell Sci. 117, 4779–86 (2004). 15331638 10.1242/jcs.01357

[b2] Lammerding J. *et al.* Lamin A/C deficiency causes defective nuclear mechanics and mechanotransduction. J. Clin. Invest. 113, 370–78 (2004). 14755334 10.1172/JCI19670 PMC324542

[b3] Guck J. *et al.* Optical deformability as an inherent cell marker for testing malignant transformation and metastatic competence. Biophys. J. 88, 3689–3698 (2005). 15722433 10.1529/biophysj.104.045476 PMC1305515

[b4] Lekka M. *et al.* Elasticity of normal and cancerous human bladder cells studied by scanning force microscopy. Eur. Biophys. J. 28, 312–316 (1999). 10394623 10.1007/s002490050213

[b5] Beckmann J., Schubert R., Chiquet-Ehrismann R. & Müller D. J. Deciphering Teneurin Domains That Facilitate Cellular Recognition, Cell−Cell Adhesion, and Neurite Outgrowth Using Atomic Force Microscopy-Based Single-Cell Force Spectroscopy. Nano Lett. 13, 2937–2946 (2013). 23688238 10.1021/nl4013248

[b6] Sylvius N. *et al.* *In vivo* and *in vitro* examination of the functional significances of novel lamin gene mutations in heart failure patients, J. Med. Genet. 42, 639–647 (2005). 16061563 10.1136/jmg.2004.023283 PMC1736117

[b7] Aebi U., Cohn J., Buhle L. & Gerace L. The nuclear lamina is a meshwork of intermediate-type filaments. Nature 323, 6088, 560–564 (1986). 3762708 10.1038/323560a0

[b8] Burke B. & Stewart C. L. The nuclear lamins: flexibility in function. Nature Reviews 14, 13–24 (2013). 10.1038/nrm3488 23212477

[b9] González Avalos P., Reichenzeller M., Eils R. & Gladilin E. Probing compressibility of the nuclear interior in wild-type and lamin deficient cells using microscopic imaging and computational modeling. J. Biomech. 44, 2642–8 (2011). 21906741 10.1016/j.jbiomech.2011.08.014

[b10] Muller D. J. AFM: A Nanotool in Membrane Biology. Biochemistry 47, 7986–7998 (2008). 18616288 10.1021/bi800753x

[b11] Martinelli V. *et al.* Carbon Nanotubes Promote Growth and Spontaneous Electrical Activity in Cultured Cardiac Myocytes. Nano Letters 12, 1831–38 (2012). 22432413 10.1021/nl204064s

[b12] Martinelli V. *et al.* Carbon Nanotubes Instruct Physiological Growth and Functionally Mature Syncytia: Nongenetic Engineering of Cardiac Myocytes. ACS Nano 7, 5746–56 (2013). 23734857 10.1021/nn4002193

[b13] Long C. S., Kariya K., Karns L. & Simpson P. C. Sympathetic Modulation of the Cardiac Myocyte Phenotype: Studies with a Cell-Culture Model of Myocardial Hypertrophy. Basic Res. Cardiol. 87 Suppl 2, 19–31 (1992). 1338564 10.1007/978-3-642-72477-0_3

[b14] Deng X. F., Rokosh D. G. & Simpson P. C. Autonomous and Growth Factor-Induced Hypertrophy in Cultured Neonatal Mouse Cardiac Myocytes. Comparison with Rat. Circ. Res. 87, 781–8 (2000). 11055982 10.1161/01.res.87.9.781

[b15] Hajjar R. J., Kang J. X., Gwathmey J. K. & Rosenzweig A. Physiological Effects of Adenoviral Gene Transfer of Sarcoplasmic Reticulum Calcium ATPase in Isolated Rat Myocytes. Circulation 95, 423–429 (1997). 9008460 10.1161/01.cir.95.2.423

[b16] Kass-Eisler A. *et al.* Quantitative determination of adenovirus-mediated gene delivery to rat cardiac myocytes *in vitro* and *in vivo*. PNAS 90, 11498–11502 (1993). 8265580 10.1073/pnas.90.24.11498 PMC48011

[b17] Kovacic-Milivojević B. *et al.* Focal adhesion kinase and p130Cas mediate both sarcomeric organization and activation of genes associated with cardiac myocyte hypertrophy. Mol. Biol. Cell 12, 2290–307 (2001). 11514617 10.1091/mbc.12.8.2290 PMC58595

[b18] Qi Y. *et al.* Moderate cardiac-selective overexpression of angiotensinII type 2 receptor protects cardiac functions from ischaemic injury. Ex.p Physiol. 97, 89–101 (2011). 10.1113/expphysiol.2011.060673 PMC3619662 21967903

[b19] Davidson P. M. & Lammerding J. Broken nuclei—lamins, nuclear mechanics, and disease Trends Cell Biol. 24, 247–56 (2014). 24309562 10.1016/j.tcb.2013.11.004 PMC3972295

[b20] Ujihara Y., Nakamura M., Miyazaki H. & Wada S. Segmentation and Morphometric Analysis of Cells from Fluorescence Microscopy Images of Cytoskeletons. Comput. Math. Methods Med. 2013, 1–11 (2011). 10.1155/2013/381356 PMC3665187 23762186

[b21] Shoji K., Ohashi K., Sampei K., Oikawa M. & Mizuno K. Cytochalasin D acts as an inhibitor of the actin-cofilin interaction. Biochem. Biophys. Res. Commun. 424(1), 52–72 (2012). 22728040 10.1016/j.bbrc.2012.06.063

[b22] Sen S., Subramanian S. & Discher D. E. Indentation and Adhesive Probing of a Cell Membrane with AFM: Theoretical Model and Experiments Biophys. J. 89, 3203–3213 (2005). 16113121 10.1529/biophysj.105.063826 PMC1366816

[b23] Lulevich V. *et al.* Cell Mechanics Using Atomic Force Microscopy-Based Single-Cell Compression. Langmuir 22, 8151–8155 (2006). 16952255 10.1021/la060561p

[b24] Landau L. D. & Lifshits E. M. Theory of Elasticity 3rd edn (Pergamon Press: Oxford; New York, 1965).

[b25] Rosenbluth M. J., Lam W. A. & Fletcher D. A. Force microscopy of nonadherent cells: a comparison of leukemia cell deformability. Biophys. J. 90, 2994–3003 (2006). 16443660 10.1529/biophysj.105.067496 PMC1414579

[b26] Sato H. *et al.* Kinetic study on the elastic change of vascular endothelial cells on collagen matrices by atomic force microscopy. Colloids Surf. B: Biointerfaces 34, 141–146 (2004). 15261083 10.1016/j.colsurfb.2003.12.013

[b27] Lieber S. C. *et al.* Aging increases stiffness of cardiac myocytes measured by atomic force microscopy nanoindentation. Am. J. Physiol. Heart Circ. Physiol. 287, H645–H651 (2004). 15044193 10.1152/ajpheart.00564.2003

[b28] Caille N., Thoumine O., Tardy Y. & Meister J. J. Contribution of the nucleus to the mechanical properties of endothelial cells. J. Biomech. 35, 177–87 (2002). 11784536 10.1016/s0021-9290(01)00201-9

[b29] Taubenberger Anna. *et al.* Revealing Early Steps of α_2_β_1_ Integrin-mediated Adhesion to Collagen Type I by Using Single-Cell Force Spectroscopy. Mol. Biol. Cell 18, 1634–1644 (2007). 17314408 10.1091/mbc.E06-09-0777 PMC1855039

[b30] Rico F. *et al.* Probing mechanical properties of living cells by atomic force microscopy with blunted pyramidal cantilever tips. Phys. Rev. E Stat. Nonlin. Soft Matter Phys. 72, 021914 (2005). 16196611 10.1103/PhysRevE.72.021914

[b31] Leporatti S. *et al.* Elasticity and adhesion of resting and lipopolysaccharide-stimulated macrophages. FEBS Lett. 580, 450–454 (2006). 16376879 10.1016/j.febslet.2005.12.037

[b32] Qin Z. & Buehler M. J. “Flaw Tolerance of Nuclear Intermediate Filament Lamina under Extreme Mechanical Deformation”. ACS Nano 5, 3034–3042 (2011). 21384869 10.1021/nn200107u

[b33] Vaziri A., Lee H. & Kaazempur Mofrad M. R. Deformation of the cell nucleus under indentation: Mechanics and mechanisms. J. Mater. Res. 21, 2126–35 (2006).

[b34] Parry D. A. D., Strelkov S. V., Burkhard P., Aebi U. & Herrmann H. Towards a molecular description of intermediate filament structure and assembly. Exp. Cell Res. 313, 2204–2216 (2007). 17521629 10.1016/j.yexcr.2007.04.009

[b35] Sokolova A. V. *et al.* Monitoring intermediate filament assembly by small-angle x-ray scattering reveals the molecular architecture of assembly intermediates. PNAS 103, 16206–16211 (2006). 17050693 10.1073/pnas.0603629103 PMC1637561

[b36] Strelkov S. V. *et al.* Divide-and-conquer crystallographic approach towards an atomic structure of intermediate filaments. J. Mol. Biol. 306, 773–781 (2001). 11243787 10.1006/jmbi.2001.4442

[b37] Strelkov S. V. *et al.* Conserved segments 1A and 2B of the intermediate filament dimer: their atomic structures and role in filament assembly. Embo J. 21, 1255–1266 (2002). 11889032 10.1093/emboj/21.6.1255 PMC125921

[b38] Lulevich V., Shih Y. P., Lo S. H. & Liu G. Y. Cell Tracing Dyes Significantly Change Single Cell Mechanics. J. Phys. Chem. B 113, 6511–6519 (2009). 19366241 10.1021/jp8103358 PMC2698996

[b39] Yang L. *et al.* Mutations in *LMNA* Modulate the Lamin A—Nesprin-2 Interaction and Cause LINC Complex Alterations. Plos One 20, e71850 (2013). 23977161 10.1371/journal.pone.0071850 PMC3748058

[b40] Crisp M. *et al.* Coupling of the nucleus and cytoplasm: role of the LINC complex. J. Cell Biol. 172, 41–53 (2006). 16380439 10.1083/jcb.200509124 PMC2063530

[b41] Cattin M. E. *et al.* Heterozygous LmnadelK32 mice develop dilated cardiomyopathy through a combined pathomechanism of haploinsufficiency and peptide toxicity. Hum. Mol. Genet. 22, 3152–64 (2013). 23575224 10.1093/hmg/ddt172

[b42] Ho C. Y., Jaalouk D. E., Vartiainen M. K. & Lammerding J. Lamin A/C and emerin regulate MKL1—SRF activity by modulating actin dynamics. Nature 497, 507–11 (2013). 23644458 10.1038/nature12105 PMC3666313

[b43] Dahl K. M. & Kalinowski A. Nucleoskelton mechanics at a glance. J. Cell Sci. 124, 675–678 (2011). 21321324 10.1242/jcs.069096 PMC3039014

[b44] Zwerger M. *et al.* Myopathic lamin mutations impair nuclear stability in cells and tissue and disrupt nucleo-cytoskeletal coupling. Hum. Mol. Genet. 22, 2335–49 (2013). 23427149 10.1093/hmg/ddt079 PMC3658163

[b45] Guilak F. Compression-induced changes in the shape and volume of the chondrocyte nucleus. J. Biomech. 28, 1529–1541 (1995). 8666592 10.1016/0021-9290(95)00100-x

[b46] Hale C. M. *et al.* Dysfunctional Connections Between the Nucleus and the Actin and Microtubule Networks in Laminopathic Models. Biophys. J. 95, 5462–5475 (2008). 18790843 10.1529/biophysj.108.139428 PMC2586579

[b47] Mounkes L., Kozlov S., Burke B. & Stewart C. L. The laminopathies: nuclear structure meets disease. Curr. Opin. Genet. Dev. 13, 223–230 (2003). 12787783 10.1016/s0959-437x(03)00058-3

[b48] Maniotis A. J., Chen C. S. & Ingber D. E. Demonstration of mechanical connections between integrins, cytoskeletal filaments, and nucleoplasm that stabilize nuclear structure. PNAS 94, 849–854 (1997). 9023345 10.1073/pnas.94.3.849 PMC19602

[b49] Stehbens W. E., Delahunt B., Shozawa T. & Gilbert-Barness E. Smooth muscle cell depletion and collagen types in progeric arteries. Cardiovasc. Pathol. 10, 133–136 (2001). 11485857 10.1016/s1054-8807(01)00069-2

[b50] Yuan C., Chen A., Kolb P. & Moy V. T. Energy landscape of the streptavidin-biotin complexes measured by atomic force microscopy. Biochemistry 39, 10219–10223 (2000). 10956011 10.1021/bi992715o

[b51] Mathur A. B., Collinsworth A. M., Reichert W. M., Kraus W. E. & Truskey G. A. Endothelial, cardiac muscle and skeletal muscle exhibit different viscous and elastic properties as determined by atomic force microscopy. J. Biomech. 34, 1545–1553 (2001). 11716856 10.1016/s0021-9290(01)00149-x

[b52] Janovjak H., Struckmeier J. & Muller D. J. Hydrodynamic effects in fast AFM single-molecule force measurements. Eur. Biophys. J. 34, 91–96 (2005). 15257425 10.1007/s00249-004-0430-3

[b53] Ohashi T., Ishii Y., Ishikawa Y., Matsumoto T. & Sato M. Experimental and numerical analyses of local mechanical properties measured by atomic force microscopy for sheared endothelial cell. BioMed. Mater. Eng. 12, 319–327 (2002). 12446947

[b54] Kuznetsova G. T., Starodubtseva M. N., Yegorenkov N. I., Chizhik S. A. & Zhdanov R. I. Atomic force microscopy probing of cell elasticity. Micron 38, 824–833 (2007). 17709250 10.1016/j.micron.2007.06.011

[b55] Weisenhorn A. L., Khorsandi M., Kasas S., Gotzos V. & Butt H. J. Deformation and height anomaly of soft surfaces studied with an AFM. Nanotechnology 4, 106–113 (1993).

[b56] Radmacher M., Fritz M. & Hansma P. K. Imaging soft samples with the atomic force microscope: gelatin in water and propanol. Biophys. J. 69, 264–270 (1995). 7669903 10.1016/S0006-3495(95)79897-6 PMC1236243

[b57] Johnson K. L., Kendall K. & Roberts A. D. Surface energy and the contact of elastic solids. Proc. R. Soc. Lond. Ser. A 324, 301–321 (1971).

[b58] Sneddon I. N. The relation between load and penetration in the axisymmetric boussinesq problem for a punch of arbitrary profile. Int. J. Eng. Sci. 3, 47–57 (1965)

[b59] Iacus S. M. & Masarotto G. in Laboratorio di statistica con R (Mc Graw-Hill Education, 2007).

